# Supplementary material for: Design of Etched- and Functionalized-Halloysite/Meloxicam Hybrids: A Tool for Enhancing Drug Solubility and Dissolution Rate
Source: Pharmaceutics. 2024 Feb 28;16(3):338. doi: 10.3390/pharmaceutics16030338 (PMC10975188; doi:10.3390/pharmaceutics16030338)
Supplement: Supplementary file 1 [file pharmaceutics-16-00338-s001.zip › pharmaceutics-2867649-supplementary.pdf]

Design of etched- and functionalized halloysite/meloxicam hybrids: a tool for enhancing drug solubility and dissolution rate

Valeria Friuli[1,*], Claudia Urru[2], Chiara Ferrara[3], Debora Maria Conti[2], Giovanna Bruni[2], Lauretta Maggi[1,*], and Doretta Capsoni[2]

[1] Department of Drug Sciences, University of Pavia, Via Taramelli 12, 27100 Pavia, Italy; valeria.friuli@unipv.it (V.F.); lauretta.maggi@unipv.it (L.M.)

[2] Department of Chemistry, Physical Chemistry Section & C.S.G.I. (Consorzio Interuniversitario per lo Sviluppo dei Sistemi a Grande Interfase), University of Pavia, 27100 Pavia, Italy; claudia.urru01@universitadipavia.it (C.U.); deboramaria.conti01@universitadipavia.it (D.M.C.); giovanna.bruni@unipv.it (G.B.); doretta.capsoni@unipv.it (D.C.)

[3] Department of Materials Science, University of Milano-Bicocca, Via Cozzi 55, 20125 Milano, Italy; chiara.ferrara@unimib.it (C.F.)

* Correspondence: valeria.friuli@unipv.it; Tel.: +39-0382-987303; lauretta.maggi@unipv.it; Tel.; +39-0382-987367

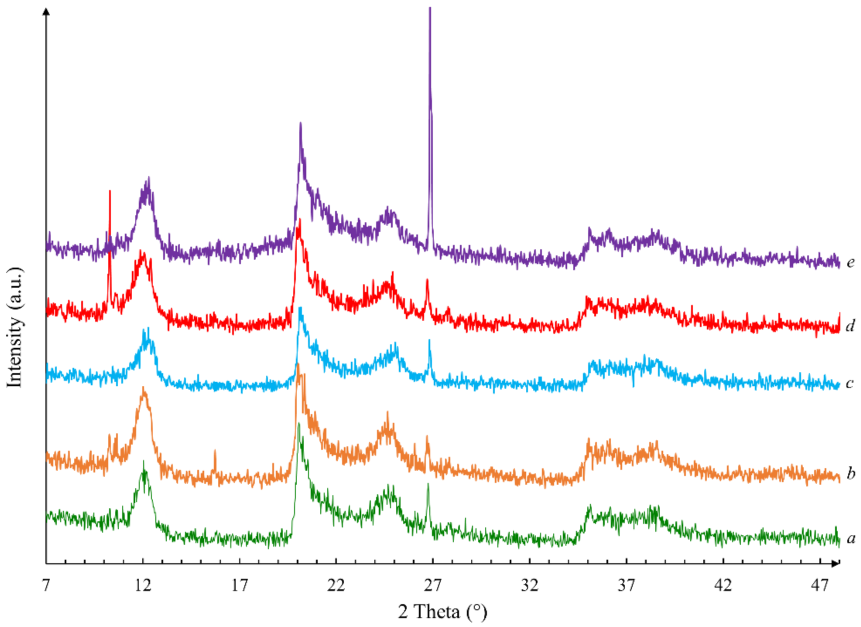

Figure S1. XRPD patterns of modified-halloysite samples: (*a*) H_HCl_2M, (*b*) H_HCl_4M, (*c*) H_NaOH_0.5M, (*d*) H_A, (*e*) H_C.

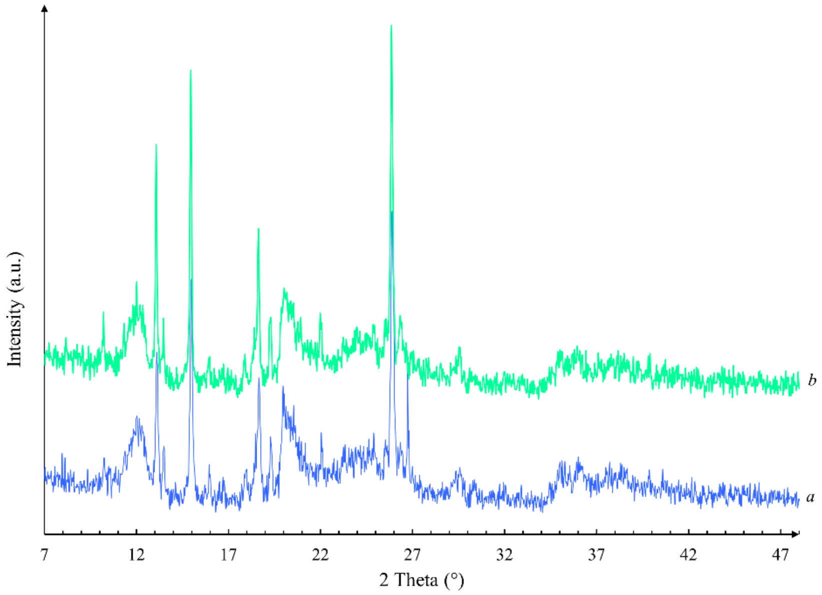

Figure S2. XRPD pattern of (*a*) MH_HCl_2M, (*b*) MH_HCl_4M.

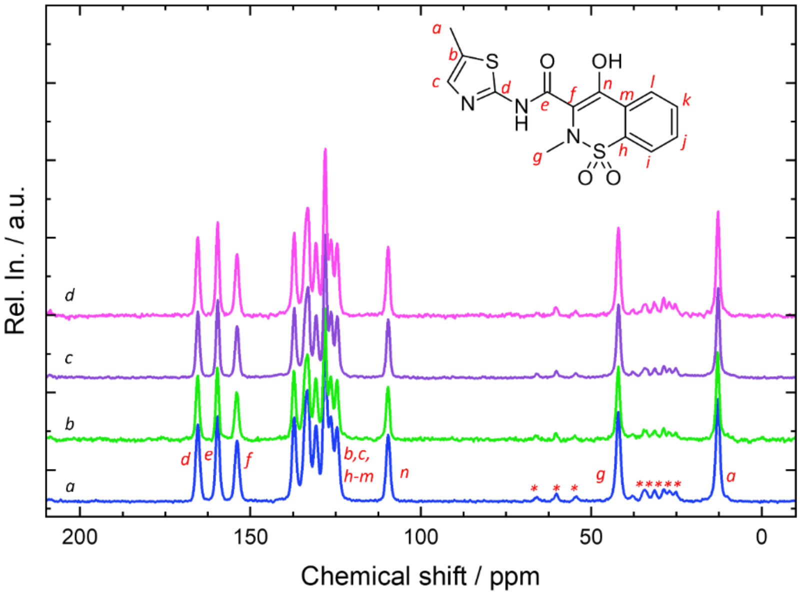

Figure S3. ^{13}C CPMAS spectra for (a) MEL, (b) MH, (c) MH-A, (d) MH-C samples and signals attribution for the MEL pristine compound.

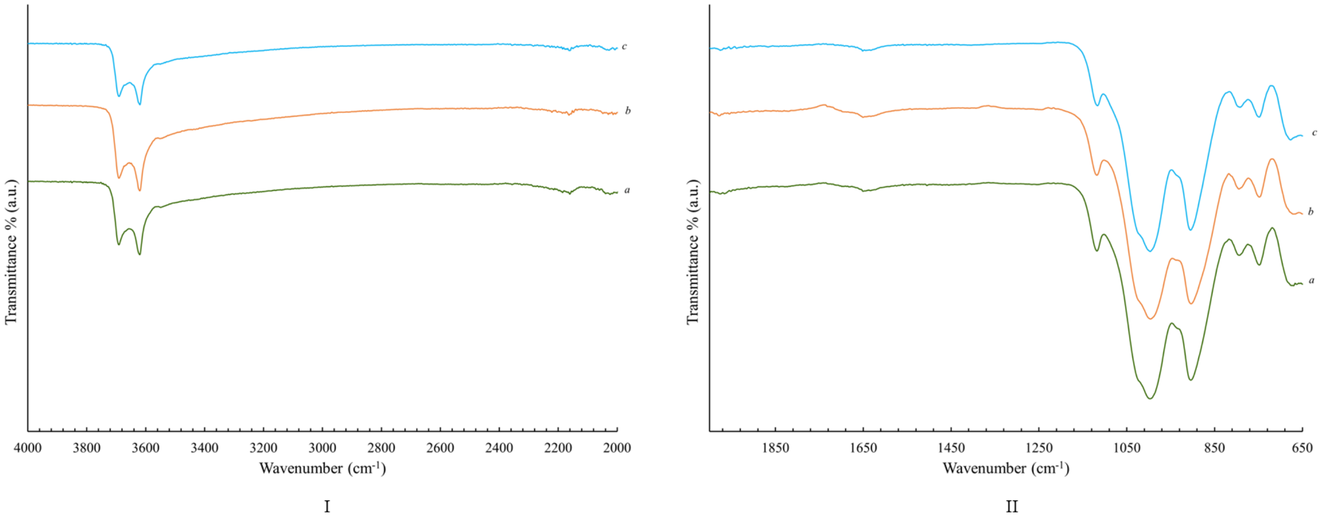

Figure S4. FT-IR spectra of (*a*) H_HCl_2M, (*b*) H_HCl_4M, and (*c*) H_NaOH_0.5M in (I) 4000 – 2000 cm^{-1}, and (II) 2000 – 650 cm^{-1} wavenumber range.

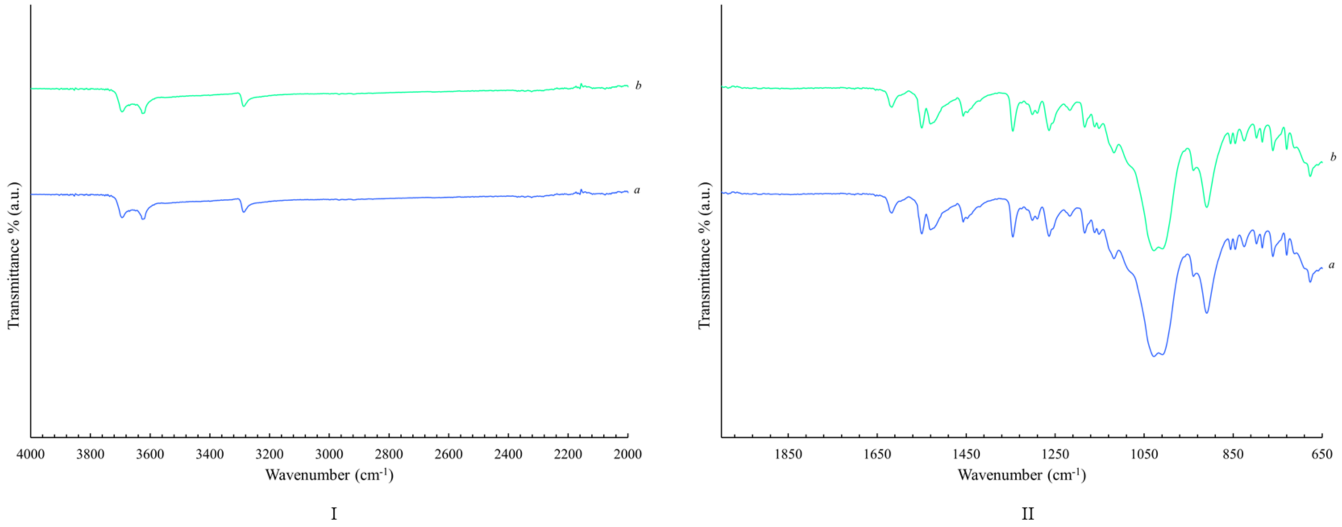

Figure S5. FT-IR spectra of (*a*) MH_HCl_2M, and (*b*) MH_HCl_4M in (I) 4000 – 2000 cm^{-1}, and (II) 2000 – 650 cm^{-1} wavenumber range.

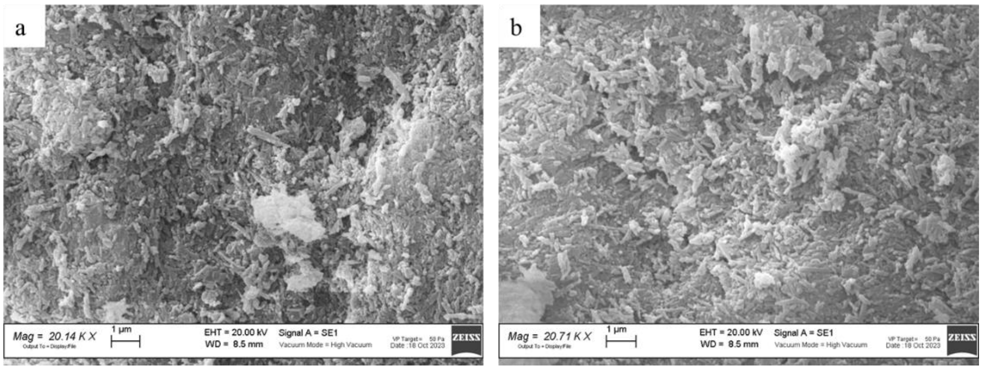

Figure S6. SEM images at 20 kX magnification of (a) H_HCl_2M, and (b) H_HCl_4M.

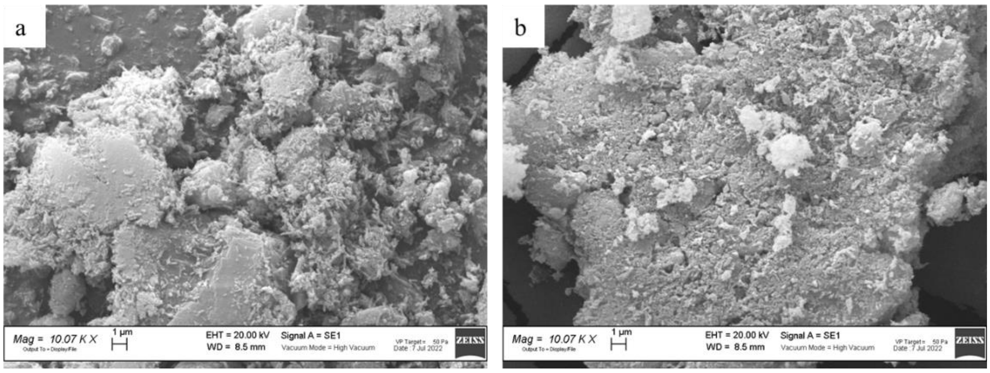

Figure S7. SEM images at 10kX magnification of (a) MH_HCl_2M, and (b) MH_HCl_4M.

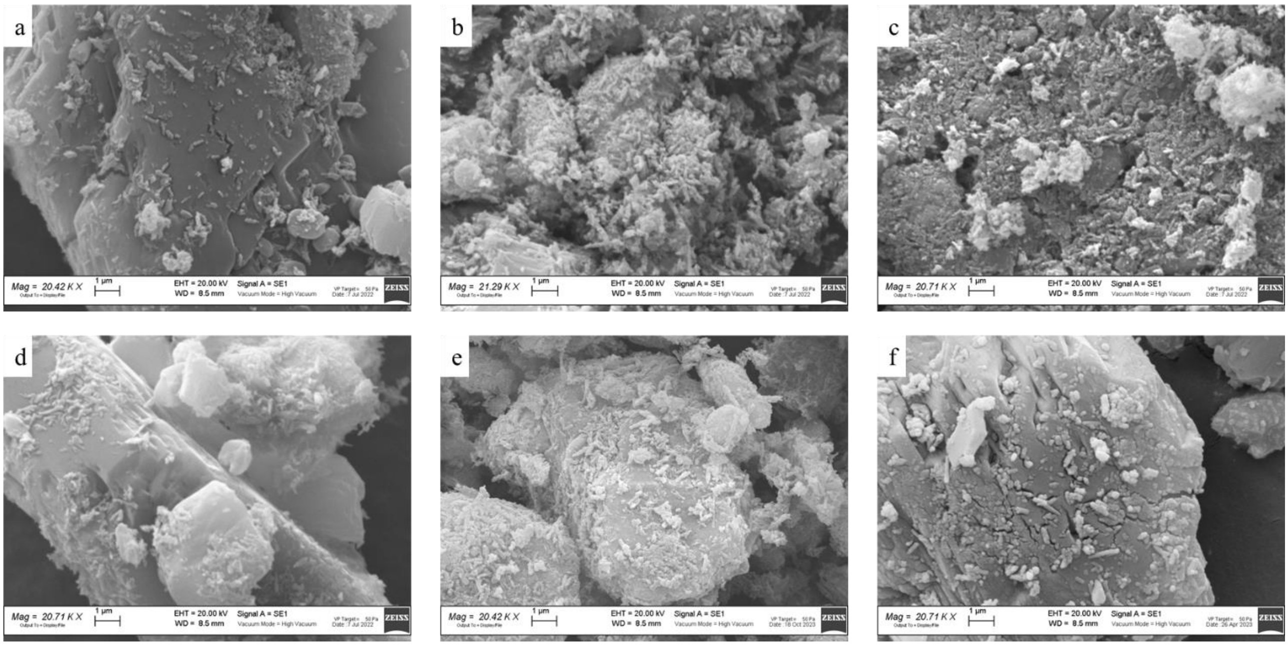

Figure S8. SEM images at 20 kX magnification of (a) MH, (b) MH_HCl_2M, (c) MH_HCl_4M, (d) MH_NaOH_0.5M, (e) MH_A and (f) MH_CTS.

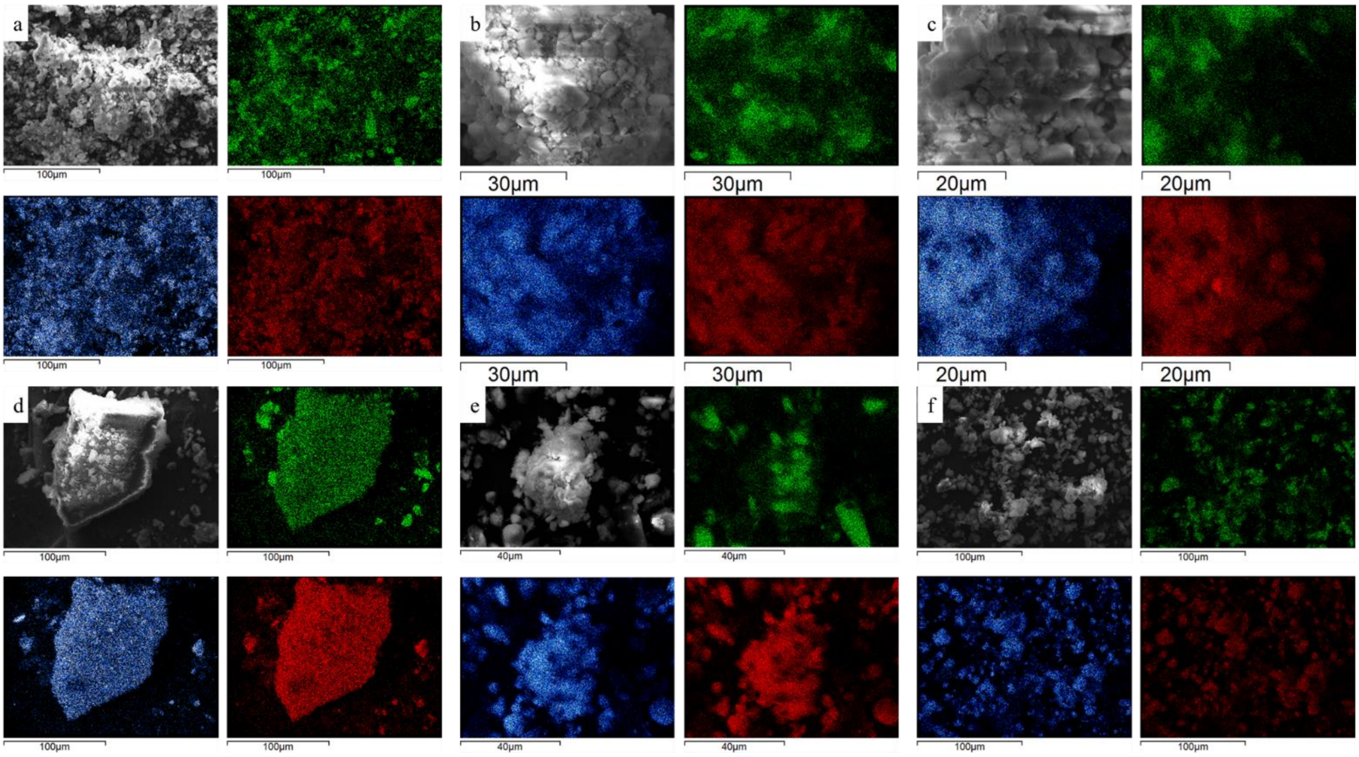

Figure S9. EDS analysis: investigated region (gray scale), S (green), Al (Blue) and Si (red) distribution maps for the (a) MH, (b) MH_HCl_2M, (c) MH_HCl_4M, (d) MH_NaOH_0.5M, (e) MH_A, (f) MH_C.

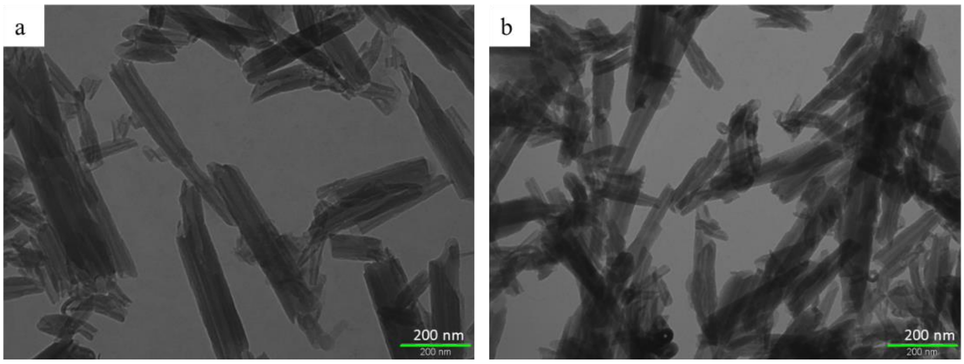

Figure S10. TEM images at 100kX magnification of (a) H_HCl_2M, and (b) H_HCl_4M.

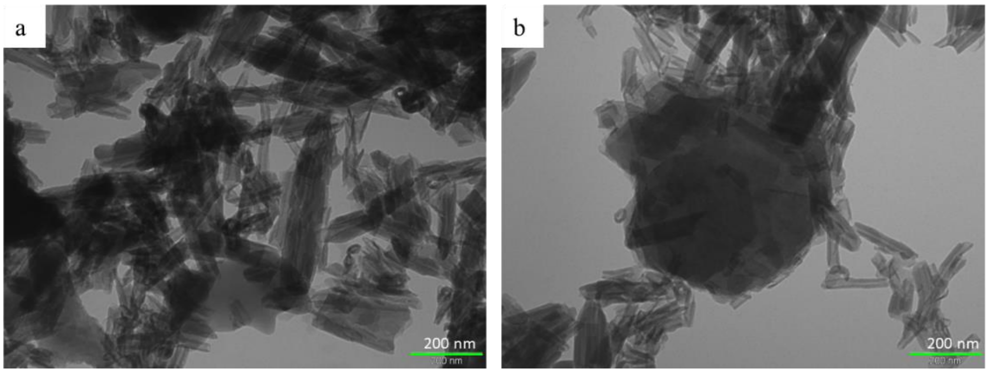

Figure S11. TEM images at 100kX magnification of (a) MH_HCl_2M, and (b) MH_HCl_4M.

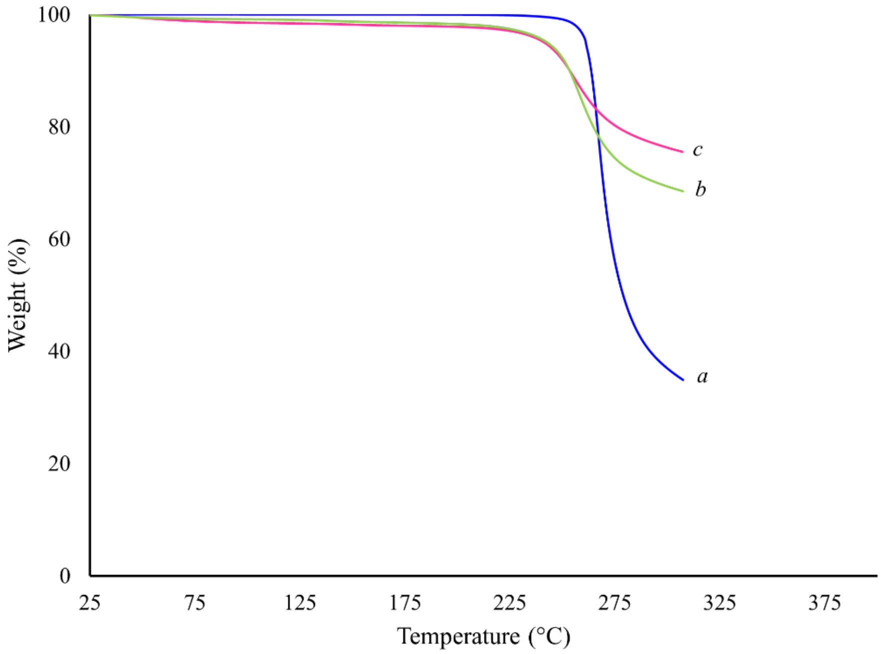

Figure S12. TG curves of (*a*) MEL, (*b*) MH, and (*c*) MH_C.

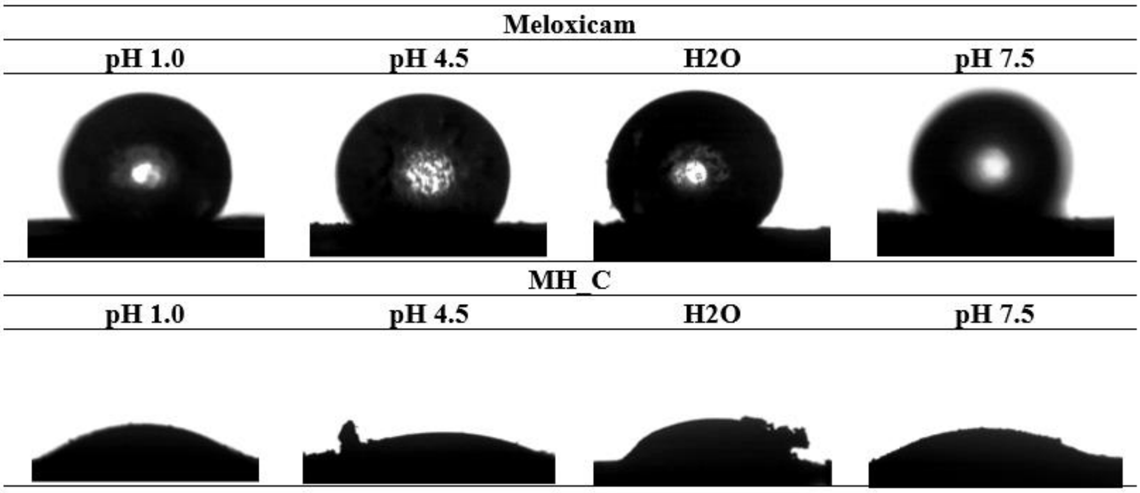

Figure S13. Contact angle test: images of MEL and MH_C drops in the different fluids at the time of 30 sec.

Table S1. Crystallite size of meloxicam in the drug-clay samples.

Sample	MEL	MH	MH_HCl_2M	MH_HCl_4M	MH_NaOH_0.5M	MH_A	MH_C
**Crystallite size (nm)**	68±3	66±2	48±4	57±3	50±4	61±2	58±3

Table S2. FT-IR bands and assignments.

Assignments	Position (cm^{-1})			
**Sample**	**H**	**MEL**	**CTS**	**APTES**
Al-OH stretching of inner-surface hydroxyl groups	3690	-	-	-
Al-OH stretching of inner hydroxyl groups	3619	-	-	-
O-H stretching of interlayer water	3545	-	-	-
O-H stretching	-	-	3360	-
N-H stretching of amidic group	-	3285	-	-
N-H stretching	-	-	3278	-
C-H stretching	-	-	-	2973<br>2927<br>2882
C-H symmetric stretching	-	-	2911	-
C-H asymmetric stretching	-	-	2865	-
C=O stretching of amide I	-	-	1651	-
O-H deformation of water	1647	-	-	-
C=O stretching of amidic group	-	1616	-	-
N-H bending of primary amine	-	-	1584	-
N-H bending of amide II	-	-	1560	-
Stretching of aromatic ring	-	1548<br>1524<br>1456<br>1446	-	-
N-H stretching and bending	-	-	-	1482<br>1440
CH_2 bending	-	-	1419	-
CH_3 symmetrical deformation	-	-	1372	-
Asymmetric stretching of SO_2 group	-	1344	-	-
C-N stretching of amide III	-	-	1316	-
Symmetric stretching of SO_2 group	-	1182	-	-
Symmetric stretching of C-O-C bridge	-	-	1151	-
Stretching vibration of C-O group	-	1118	-	-
Si-O stretching	1117	-	-	-
C-O stretching	-	-	1062<br>1027	-
Stretching vibration of C-N group	-	1042	-	-
Si-O-Si stretching	1011	-	-	-
Al-O-H bending	902	-	-	-

Table S3. Meloxicam weight percentage in drug-clays systems evaluated by EDS microanalysis.

Sample	MH	MH_HCl_2M	MH_HCl_4M	MH_NaOH_0.5M	MH_A	MH_C
**Meloxicam (wt%)**	40.0±3.2	35.4±4.4	31.0±4.1	40.0±2.7	36.2±3.7	42.5±4.1
